# Circumferential electrocautery of the patella in primary total knee replacement without patellar replacement: a meta-analysis and systematic review

**DOI:** 10.1038/srep09393

**Published:** 2015-03-24

**Authors:** Lihong Fan, Zhaogang Ge, Chen Zhang, Jia Li, Zefeng Yu, Xiaoqian Dang, Kunzheng Wang

**Affiliations:** 1 Department of Orthopedics, The Second Affiliated Hospital of Xi'an Jiaotong University, Xiwu Road, Xi'an, Shaanxi 710004, China

## Abstract

The purpose of this meta-analysis and systematic review was to identify and assess whether circumferential electrocautery is useful for improving outcomes after primary total knee replacement(TKR). We searched MEDLINE, EMBASE, PubMed, SpringerLink, Web of Knowledge, OVID CINAHL, OVID EBM and Google Scholar and included articles published through January 2014. A total of 6 articles met the inclusion criteria. Of the 776 cases included in the analysis, 388 cases involved patellar denervation, and 388 cases were designated as the control group. The meta-analysis revealed no significant difference in the incidence of anterior knee pain (AKP, p = 0.18) or in the visual analogue scale score (VAS, p = 0.23) between the two groups. In addition, AKSS Function Score indicated no significant difference between the two groups (p = 0.28). However, the OKS (p = 0.02), patellar score (p = 0.01), AKSS-Knee Score (p = 0.004), range of motion (ROM, p < 0.0001) and WOMAC Score (p = 0.0003) indicated that circumpatellarelectrocautery improved clinical outcomes compared with non-electrocautery. The results indicate that circumferential electrocautery of the patella does not significantly improve AKP compared with non-electrocautery techniques but that circumferential electrocautery significantly improves patients' knee function after surgery. Therefore, we believe that circumferential electrocautery is beneficial to the outcome of primary TKR surgery without patellar replacement.

The treatment of the patella during primary total knee replacement (TKR)continues to be debated. However, many studies^1^,^2^,^3^,^4^,^5^,^6^,^7^ have indicated that patellar replacement in primary TKR does not improve the outcome. Thus, certain orthopaedic surgeons perform primary TKR surgery without patellar replacement^7^,^8^. Determining the best way to improve the outcome of primary TKR is a significant clinical problem.

One of the most important problems after the surgery is anterior knee pain (AKP). Many measures have been used to solve this problem, including resurfacing; however, certain studies have indicated that patellar resurfacing does not improve outcomes after primary TKR. One of these studies^9^ included a pragmatic, multicentre, randomized controlled trial (RCT) of 1715 patients. In this large RCT of patellar resurfacing, the functional outcome and reoperation rate five years after primary TKA were not significantly affected by the addition of patellar resurfacing to the surgical procedure.

However, Vega, Golano and Perez-Carro^10^ described a technique that involves a thermal lesion applied to the peripatellar soft tissues to treat AKP. This technique of circumferential electrocautery is inserted through suprapatellar approaches to produce a thermal lesion in the peripatellar synovial tissue, thereby reducing the level of pain signals. This technique should in turn reduce the severity and incidence of AKP. With increasing numbers of TKRs and the importance of patient expectations, addressing the problem of AKP after TKR is of special significance. Whether circumferential electrocautery is useful for improving outcomes after primary TKR is controversial. To date, we are aware of only 6 studies, and these studies have drawn opposite conclusions. In addition, only one meta-analysis has been performed.

The purpose of the present meta-analysis and systematic review was therefore to identify and assess whether circumferential electrocautery is useful for improving outcomes after the primary TKR surgery without patella replacement. In addition, we also sought to determine whether circumferential electrocautery is an ideal choice in primary TKR surgery.

## Results

**Figure 1:**
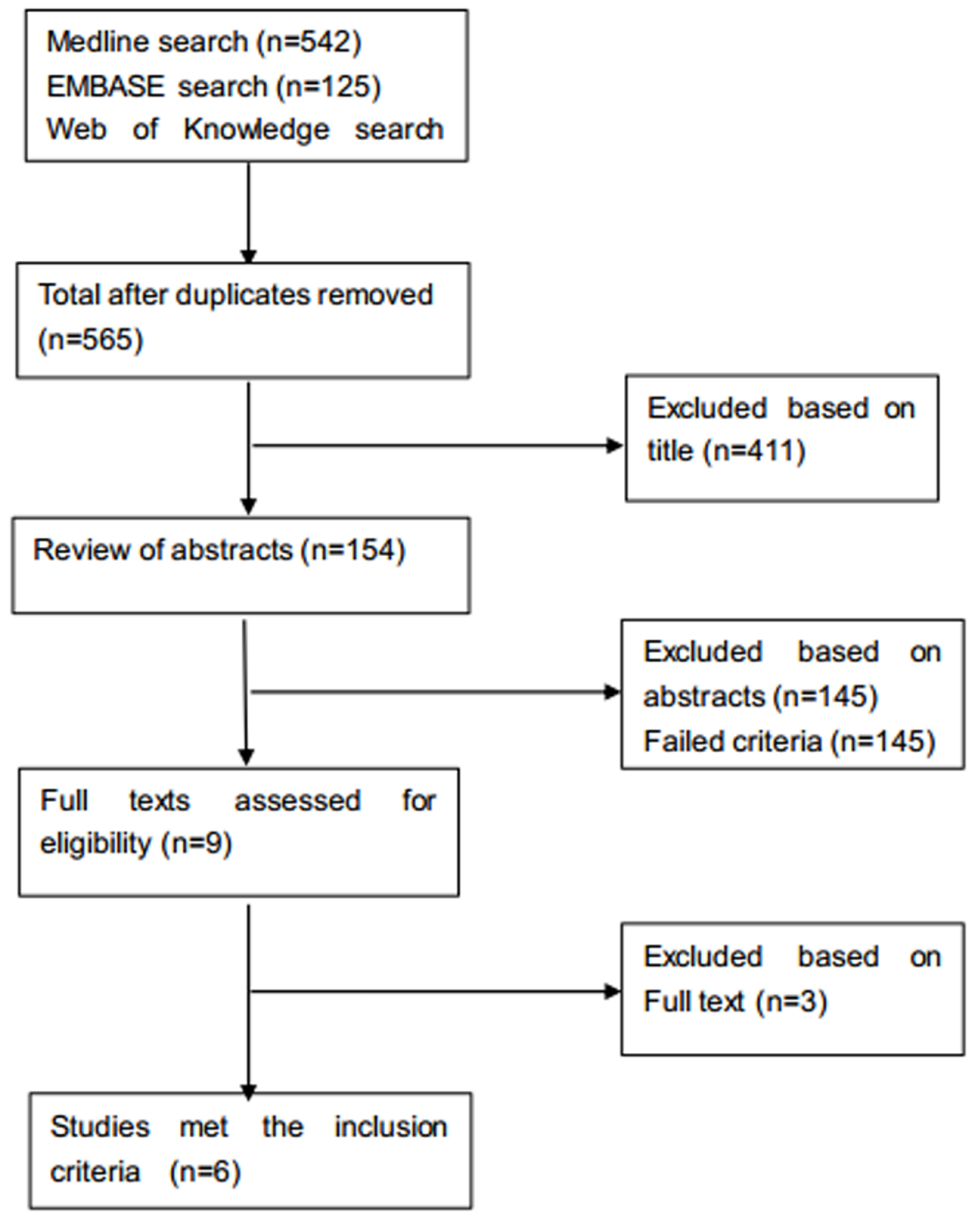
Flowchart illustrating the literature search.

A total of 6 studies^11^,^13^,^14^,^15^,^17^,^18^ met the inclusion criteria. Of the 776 cases in total, 388 cases involved patellar denervation, and 388 cases were designated as the control group, as indicated by the flowchart in Figure 1. The main characteristics and the quality of the included studies according to the Modified Jadad Score (7-points) are reported in Tables 1 and 2, respectively.

The characteristics of these 6 studies are presented in Table 1. The demographics of each group were not significantly different in terms of the main characteristics. Statistically significant differences in the pre-operative outcome measures of these studies were not observed pre-operatively between the groups. All patients underwent standard TKR with either a low contact stress (LCS) system or the Kinemax (Zimmer, Warsaw, Indiana) systems. Two studies investigated patients undergoing bilateral TKA^15^,^17^ with electrocautery denervation of one patella and no denervation of the other. The follow-up period lasted for at least 9 months. The outcome measures of these studies included the incidence of AKP^11^,^13^,^14^,^17^,^18^, VAS^11^,^15^,^18^, AKSS^13^,^15^,^17^,^18^, OKS^11^,^18^, patellar score^15^,^17^,^18^, ROM^15^,^17^,^18^, WOMAC Score^13^,^17^ and other measures. We used data reporting a change from baseline as our effect index.

Two^13^,^18^ of the six included studies described randomization (using computer-generated random numbers), and four studies^11^,^13^,^15^,^18^ used adequate concealment of allocation (in an opaque, sealed envelope) and a double-blind method (observer and patient blinding). The methodological quality of the included studies was assessed using the Modified Jadad Score (7-points, as shown in Table 2).

Five of the six studies provided data on the incidence of AKP^11^,^13^,^14^,^17^,^18^. The overall incidence of AKP in this study is 38.6%. In addition, 34.9% (123/352) of the electrocauterized knees experienced AKP, compared with 42.3% (149/352) of the non-electrocauterized knees (RR = 0.78, 95% CI (0.55–1.12), I^2^ = 70%, p = 0.18). A visual analogue scale (VAS) score was used to assess post-operative AKP in three studies^11^,^15^,^18^. The results of the studies^13^,^14^ indicated statistically significant differences between the two groups. However, the meta-analysis revealed no statistically significant difference in the VAS score between the two groups (p = 0.23).

**Figure 2:**
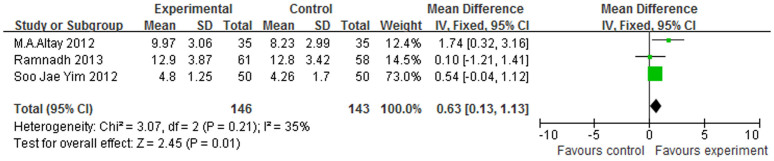
Forest plot of Patellar Scores between the circumpatellarelectrocautery and non-electrocautery groups.

**Figure 3:**
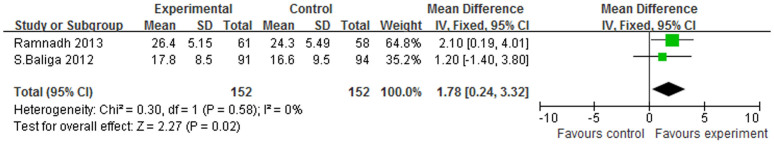
Forest plot of OKS between the circumpatellarelectrocautery and non-electrocautery groups.

**Figure 4:**
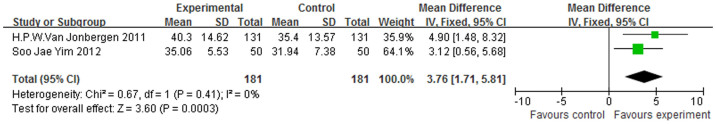
Forest plot of WOMAC between the circumpatellarelectrocautery and non-electrocautery groups.

**Figure 5:**
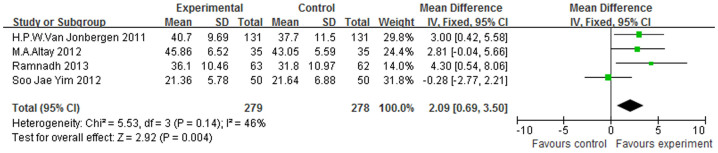
Forest plot of AKSS-Knee Scores between the circumpatellarelectrocautery and non-electrocautery groups.

Three studies included in this meta-analysis used the patellar score^15^,^17^,^18^ as their outcome measure, and we found that the electrocautery group displayed significantly better scores than those of the non-electrocautery group (WMD = 0.63, 95% CI (0.13–1.13), I^2^ = 35%, p = 0.01) (Figure 2). With regard to the OKS (Figure 3), only two studies^11^,^18^ provided relevant data, and the meta-analysis revealed statistically significant differences between the two groups (WMD = 1.78, 95% CI (0.24–3.32), I^2^ = 0%, p = 0.02). Significant differences in the WOMAC^13^,^17^ were observed between the electrocautery and the non-electrocautery TKA groups among the studies in this analysis (WMD = 3.76, 95% CI (1.71–5.81), I^2^ = 0%, p = 0.0003), (Figure 4). The AKSS^13^,^15^,^17^,^18^ includes a Knee Score and a Function Score. With regard to the Knee Score (Figure 5), the p-value was 0.0004 (WMD = 2.09, 95% CI (0.69–3.50), I^2^ = 46%). However, the meta-analysis of the Function Score indicated no statistically significant difference between the electrocautery and the non-electrocautery groups (WMD = 1.93, 95% CI (−1.57–5.43), I^2^ = 80%, p = 0.28).

**Figure 6:**
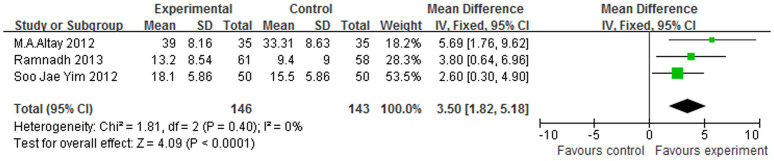
Forest plot of ROM between the circumpatellarelectrocautery and non-electrocautery groups.

The ROM value reflects the motion of the knee joint in patients after surgery. Three studies^15^,^17^,^18^ provided these data. We report that the ROM of patients in the electrocautery group was better than that of control group, with a p-value of p < 0.0001 (WMD = 3.5, 95% CI (1.82–5.18), I^2^ = 0%) (Figure 6).

We believe that no score can replace patient satisfaction. After surgery, better patient satisfaction is expected. Only one^18^ of the six studies provided this type of data, which was measured as excellent, good, fair or poor. Patient satisfaction was higher in the denervation group with more patients rating the procedure as excellent p < 0.05). With regard to complications, only three studies^13^,^14^,^18^ included described the post-operative complications in patients during short-term follow-up. The meta-analysis results indicate no statistically significant difference between the two groups (RR = 2.30, 95% CI (0.61–8.63), I^2^ = 0%, p = 0.22).

## Discussion

The most important finding of the present study was that certain studies^11^,^12^,^17^ found that circumferential electrocautery of the patella could not improve the outcome after surgery, whereas other studies^13^,^14^,^15^,^16^,^18^ found that the technique was very effective. The ultimate goal of TKR is to relieve pain and to improve the functional outcome. AKP is reported to occur in up to one-half of all patients following primary TKR. The presence of AKP after TKR is negatively correlated with patient satisfaction and quality of life. Therefore, how to improve outcomes after primary TKR without patellar replacement is a significant clinical problem.

Various methods have been attempted, such as patelloplasty, patellar resurfacing and others, but the results were controversial^1^,^2^,^3^,^4^,^5^,^7^,^8^,^9^,^19^,^20^,^21^,^22^,^23^. Certain researchers^7^,^8^,^20^,^21^,^22^,^23^ believe that resurfacing may improve outcomes after primary TKR. However, Pavlou et al.^3^ designed a meta-analysis of 7075 cases (3463 in the resurfacing group and 3612 in the non-resurfacing group), and the reoperation rates, the incidence of AKP and functional scores were used as outcome measures. However, no evidence was found to suggest that either patellar resurfacing or prosthetic design affects the clinical outcome of a total knee arthroplasty. In addition, one randomized prospective trial with a minimum of a 7-year follow-up^4^ included 133 patients observed no significant difference between the groups treated with patellar reshaping and patellar resurfacing with regard to the KSS, AKP rate and radiographs.

These conclusions^1^,^2^,^4^,^5^,^6^ might prompt us to choose primary TKP surgery without patella replacement. Moreover, given that Asian patellae are characteristically thin, exists a major risk for patellar replacement. Therefore, we tend to choose the primary TKP surgery without patellar replacement. However, how can we improve outcomes after primary TKP?

Vega and Golano^10^ surmised that a thermal lesion applied to this region would lead to desensitization of the anterior knee area in a process known as patellar denervation. Circumpatellarelectrocautery was performed using a standard technique with monopolar diathermy set at 50 W, and the synovial soft-tissue layer within 1 cm of the circumference of the patella was cauterized. The technique used only superficial electrocautery to a depth of no more than 1–3 mm^1^,^5^. We believe that this technique is theoretically feasible. One Dutch study^24^ revealed that 56% of orthopaedic surgeons performing TKA use circumpatellarelectrocautery when not resurfacing the patella and that 32% use diathermy when resurfacing the patella. Electrosurgical arthroscopic patellar denervation has been used to provide effective treatment for patients with intractable patellofemoral pain.

One meta-analysis study^25^ of circumferential electrocautery was published in June 2013. The authors found no strong evidence either for or against electrocautery compared with non-electrocautery in TKAs. Compared with this study, our study is more comprehensive. We compiled all of the data that the included studies have mentioned and discussed all of measures of outcomes after surgery. Moreover, we described our methods clearly, and showed our meta-analysis results in the form of funnel plots and figures. More importantly, we found two additional studies to data on this topic. All of the above have helped us to achieve a more scientific article.

Similar to the previous study, our meta-analysis results indicate no significant difference in the incidence of AKP or in the VAS between the two groups. However, our study displays strong evidence suggesting that circumferential electrocautery can improve several types of scores and patient satisfaction, thereby indicating that circumferential electrocautery of the patellar can significantly improve patients' knee function after TKR. Moreover, based on the studies included, every outcome measure was improvement. With regard to the results for AKP, which is influenced by numerous influencing factors, we believe that the subjectivity of pain, the different operating techniques of surgeons and racial disparities may have had a great influence on these results. In addition, the results of prior studies^13^,^14^ indicate significant differences between the two groups. Therefore, we believe that circumferential electrocautery is beneficial to the outcome of primary TKR surgery without patellar replacement.

However, to implement more effective circumferential electrocautery procedures, a better understanding of the nerve distribution around the patella will provide the opportunity for clinicians to perform effective and selective denervation to treat severe patellofemoral joint problems. Several anatomic studies^26^,^27^,^28^ investigating patellar innervation have shown that the patellar terminal branches are not uniform and that they may have a widely varied distribution. This anatomic variability is evidenced at the medial and particularly at the lateral margins of the patella. The findings of R. Shane Barton^27^ indicated that the greatest density of ION occurs within the medial and central patella that considerably less nerve tissue is observed laterally. An anatomical and clinical study^28^ found that two nerves reach as far as the patellar edge in the superomedial and superolateral quadrants, coursing within the substance of the vastusmedialis and lateralis. The topographic anatomy of the nerves varies with respect to the patellar edge. Although certain branches from the fibular nerve ascend towards the patellar tendon and fat pad, these branches could not be traced as far as the inferior patellar edge. Thus, certain researchers suggest that if denervation is preferred, the procedure should selectively include both the medial and the lateral nerves^29^. Moreover, a reasonable alternative would be to achieve denervation by producing lesions on the pain receptors located in the peripatellar soft tissue, as indicated in a study by Wotjys.

The studies included in this analysis are RCTs, but each study has limitations. We believe that too few subjects were used in these studies to inform a new principle of treatment. Moreover, a follow-up period of one year may be too short; differences between the groups may become apparent at later stages. In our study, I^2^ was >50% in the meta-analysis of the AKP incidence, the VAS and the AKSS-Function Score. From the original data, we found that heterogeneity may come from population characteristics, such as the mean age, gender, the mean pre-operative duration of symptoms, and the mean duration of follow-up. However, due to the limitation of a lack of studies on this topic, we cannot perform further analysis by subgroup analysis or other methods. More large RCTs are needed to prove that circumferential electrocautery is useful for improving outcomes after primary TKR. In addition, we should consider the nerve distribution around the patella and how to perform the operation to obtain improved outcomes.

In summary, although the conclusions of several RCTs are still controversial, the meta-analysis of these studies indicates that circumferential electrocautery of the patella does not significantly improve AKP compared with non-electrocautery but that circumferential electrocautery can improve patients' knee function after surgery. With regard to the AKP results, we believe that the subjectivity of pain, the different operating procedures of surgeons, racial disparities and the length of follow-up may significantly influence AKP. Therefore, we believe that circumferential electrocautery is beneficial to the outcome of primary TKR surgery without patellar replacement. More large RCTs from multiple centres that are scientifically designed to examine whether circumferential electrocautery is useful for improving outcomes after primary TKR without patellar replacement are needed.

## Methods

### Identification of studies

An independent researcher performed the literature search using the following search terms with Boolean operators: anterior knee pain, patellofemoral pain, retropatellar circumferential electrocautery, electrocautery, denervation, patellar denervation, patellar, patellar resurfacing, AKP, pain and knee arthroplasty, primary total knee replacement, knee replacement, total knee replacement, patelloplasty, total knee arthroplasty, TKA, TKR and TKP. Search queries were limited to the title and abstract, and the language was restricted to English. The electronic search involved the Cochrane Database of Systematic Reviews, the Cochrane Central Register of Controlled Trials, MEDLINE, EMBASE, SpringerLink, Web of Knowledge, OVID CINAHL, OVID EBM and Google Scholar and included articles published through January 2014.

### Assessment of study eligibility

Only published, full-text, peer-reviewed studies of circumferential electrocautery of the patellar in primary TKR without patellar replacement were included. Studies not reporting the prevalence of circumferential electrocautery of the patella were excluded. Moreover, publications with incompletely described patient populations, less than 10 included patients, or less than6 months of follow-up and studies that failed to describe the method used to assess the prevalence of circumferential electrocautery of the patellar were excluded. Two reviewers (Lihong Fan and Zhaogang Ge) independently examined all titles and abstracts and selected the studies for full-text review. If disagreements arose, a third investigator helped to resolve the problem. For studies with discrepancies, the authors discussed the discrepancies to reach a consensus. Additionally, the reference lists in the included studies were hand-searched for additional relevant studies. The full texts were retrieved and further examined regarding the inclusion and exclusion criteria.

### Data extraction

Data extraction was performed by one author (Zhaogang Ge) and validated by a second author (Lihong Fan). For all studies selected for full-text review, relevant data were abstracted from the text, figures, and tables using a structured data abstraction form. The following data were extracted from each study: the first author's last name, the publication year, the country where the study was conducted, the study design, the length of follow-up, the number of subjects, the outcome measures, and the means and variables studied in relation to circumferential electrocautery of the patella. If the reported data were incomplete, the corresponding authors were contacted by email to obtain additional data. Moreover, two authors (Lihong Fan and Zhaogang Ge) assessed the methodological quality of the included studies independently using the Modified Jadad Score. This 7-point assessment includes the following categories: randomization, concealment of allocation, double blinding, withdrawals and dropouts.

### Statistical analysis

The data from the included studies were tabulated to determine whether circumferential electrocautery is useful for improving outcomes after primary TKR. The risk ratio (RR) and 95% confidence interval (CI) were calculated for dichotomous data. Continuous data were assessed using the weighted mean difference (WMD) method. In addition, we used data representing a change from baseline as our effect index. A fixed-effects model was established using the inverse-variance method for continuous variables and the Mantel-Haenszel method for dichotomous variables. Statistical heterogeneity was evaluated using a standard χ2 test at a significance level of p < 0.1 and the I^2^-statistic, which describes the proportion of variability due to heterogeneity. The meta-analysis was performed using Review Manager 5.0 for measuring the outcomes, and a p-value of <0.05 was considered statistically significant.

## Author Contributions

L.F. and Z.G. conducted the literature search and determined studies for exclusion and inclusion. L.F., Z.G., C.Z., J.L., Z.Y. and X.D. extracted data from the retrieved studies, performed the meta-analysis, and drafted the manuscript. K.W. conceived the idea of the study, designed the study, and critically revised the manuscript for important intellectual content. All authors reviewed the paper and approved the final manuscript.

## Figures and Tables

**Table 1:** The main characteristics of the included studies

Ref (#)	Study Id (year)	Country	study design	follow-up	Number of electrocautery group	Number of non-electrocautery group	Outcome measures	Loss to Follow-up
1	S. Baliga (2012)	England	RCT	1 year	91	94	OKS and VAS	15
2	H. P. W. van Jonbergen (2011)	Netherlands	RCT	1 year	131	131	the incidence of anterior knee pain, WOMAC score, AKSS: knee scores and function scores	0
3	Abdelfattah Mohammed Fathy Saoud (2004)	Egypt	RCT	9 months	20	20	AKSS: knee scores and function scores	2
4	M.A. Altay (2012)	Turkey	RCT	2 years	35	35	AKSS: knee scores and function scores, patellar score, VAS and range of motion(ROM)	0
5	Soo Jae Yim (2012)	Korea	RCT	1 year	50	50	Range of Motion, AKSS: knee scores and function scores, Patellar Score, WOMAC score	0
6*	Ramnadh S. Pulavarti (2013)	England	RCT	2 year	61	58	Range of Motion, AKSS: knee scores and function scores, Patellar Score, VAS, OKS	7

OKS: Oxford knee score. VAS: visual analogue scale. AKP: anterior knee pain. AKS: American Knee Society. WOMAC: Western Ontario and McMaster Universities osteoarthritis index. RCT: randomized controlled trial.

*N = 63 in electrocautery group and N = 62 in non-electrocautery group at 12 months of follow-up.

**Table 2:** Methodological quality of the included studies by using the Modified Jadad Score (7-points)

Ref (#)	Study Id (year)	Randomization	Concealment of allocation	Double blinding	Withdrawals and dropouts	Jadad Score	quality
1	S. Baliga (2012)	Unclear	Yes	Yes	Yes	6	High
2	H. P. W. van Jonbergen (2011)	Yes	Yes	Yes	Yes	7	High
3	Abdelfattah Mohammed Fathy Saoud (2004)	Unclear	Not	Not	Yes	2	Low
4	M.A. Altay (2012)	Unclear	Yes	Yes	Yes	6	High
5	Soo Jae Yim (2012)	Unclear	Not	Not	Yes	2	Low
6	Ramnadh S. Pulavarti (2013)	Yes	Yes	Yes	Yes	7	High
